# A comparison of semi-quantitative methods suitable for establishing volatile profiles

**DOI:** 10.1186/s13007-018-0335-2

**Published:** 2018-08-09

**Authors:** Victoria Ruiz-Hernández, María José Roca, Marcos Egea-Cortines, Julia Weiss

**Affiliations:** 1 Instituto de Biotecnología Vegetal, Universidad Politécnica de Cartagena, Cartagena, Spain; 2 Departamento de Ciencia y Tecnología Agraria, Universidad Politécnica de Cartagena, Cartagena, Spain; 3 Servicio de Apoyo a la Investigación Tecnológica, Universidad Politécnica de Cartagena, Cartagena, Spain

**Keywords:** GC–MS, HSSE, Internal standard, Calibration curve, Quantification, Scent profile, Stir bars, Twisters, VOCs

## Abstract

**Background:**

Full scent profiles emitted by living tissues can be screened by using total ion chromatograms generated in full scan mode and gas chromatography–mass spectrometry technique using Headspace Sorptive Extraction. This allows the identification of specific compounds and their absolute quantification or relative abundance. Quantifications ideally should be based on calibration curves using standards for each compound. However, the unpredictable composition of Volatile Organic Compounds (VOCs) and lack of standards make this approach difficult. Researchers studying scent profiles therefore concentrate on identifying specific scent footprints i.e. relative abundance rather than absolute quantities. We compared several semi-quantitative methods: external calibration curves generated in the sampling system and by liquid addition of standards to stir bars, total integrated peak area per fresh weight (FW), normalized peak area per FW, semi-quantification based on internal standard abundance, semi-quantification based on the nearest *n*-alkane and percentage of emission. Furthermore, we explored the usage of nearest components and single calibrators for semi-quantifications.

**Results:**

Any of the semi-quantification methods based on a standard produced similar or even identical results compared to quantification by a true-standard for a compound, except for the method based on standard addition. Each method beholds advantages and disadvantages regarding level of accuracy, experimental variability, acceptance and retrieved quantities.

**Conclusions:**

Our data shows that, except for the method of standard addition to the biological sample, the rest of the semi-quantification methods studied give highly similar statistical results. Any of the methodologies presented here can therefore be considered as valid for scent profiling. Regarding relative proportions of VOCs, the generation of calibration curves for each compound analysed is not necessary.

**Electronic supplementary material:**

The online version of this article (10.1186/s13007-018-0335-2) contains supplementary material, which is available to authorized users.

## Background

The emission of Volatile Organic Compounds (VOCs) is a biological feature of bacteria, fungi, plants and animals. They play a key role in interaction between individuals of the same and other species, genera and kingdoms [1–3]. The number of identified VOCs emitted in nature is constantly increasing as the analytical techniques improve and biodiversity is scrutinized for its chemical diversity. Numerous ecological studies are focusing on VOC functions i.e. the mediation of plant defence by volatile compounds in plant communities [4].

VOCs emission by plants can be very variable, especially in flowers where different compounds comprising a specific scent profile may be counted in dozens [5]. Bioactivity of VOCs emitted by flowers is diverse and not fully understood yet. While some of these compounds are known to have an effect over pollinator attraction, others may act as repellents [6, 7].

In order to identify as many compounds as possible in a given sample, researchers use HSSE, and GC–MS using TICs when screening scent profiles. A scent profile is understood as the combination and proportion of VOCs which is conserved for a certain set of samples and reflect a combination of the genotype and environmental conditions.

The composition of floral scent profiles is robust for a given species [8]. Most scientists distinguish major and minor VOCs, where major compounds are emitted in higher quantities and can be interpreted as the characteristic footprint of a species. Minor compounds contribute to the fine tuning of the ultimate bouquet. The VOCs emitted by a plant organ changes depending on the time of the day, physiological stage and biotic and abiotic external factors [9–11].

An appropriate quantification of compounds ideally requires the use of a specific standard for each compound present in the sample [12, 13]. Considering that plant VOCs may be present in dozens, their quantifications based on the inclusion of standards for each VOC increases the economic costs considerably [14]. An additional problem is that pure standards may not be available for most compounds [15, 16]. Furthermore, while some scent profiles are composed of VOCs emitted constitutively, other compounds are emitted only under certain circumstances, and the appropriate standards are therefore not known a priori [17]. Finally, there are VOCs that are known for having several isomers with differing Chemical Abstract Service number and therefore a potential standard. As a result, performing appropriate quantifications of VOCs emitted by plants is not straightforward.

Due to the high costs and lack of standards for every chemical compound, other methods that allow semi-quantification of compounds can be used. The most common method is the usage of single internal standards for extrapolating quantities [10, 18–20]. Moreover, several internal standards can be used for semi-quantifying nearest or similar components within the chromatogram [15, 21]. However, the use of internal standards has some drawbacks related to the response of VOCs during the chromatographic analysis, such as differential binding to the adsorption fiber [22, 23].

Here we compared external calibrators obtained by adding standards to the sampling system, external calibrators by liquid addition to stir bars, calculation of integrated peak area·gFW^−1^, calculation of normalized peak area·gFW^−1^, semi-quantification based on internal standard, semi-quantification based on external calibrator area using the NearestRT *n*-alkane. Our results indicate that except for the semi-quantification by standard addition to the biological sample, the rest of the methods studied give highly similar statistical results. Furthermore, results indicate that the use of a standard for each VOC analysed in the context of scent profiles studies can be omitted.

## Methods

### Plant material and VOCs collection

**Fig. 1:**
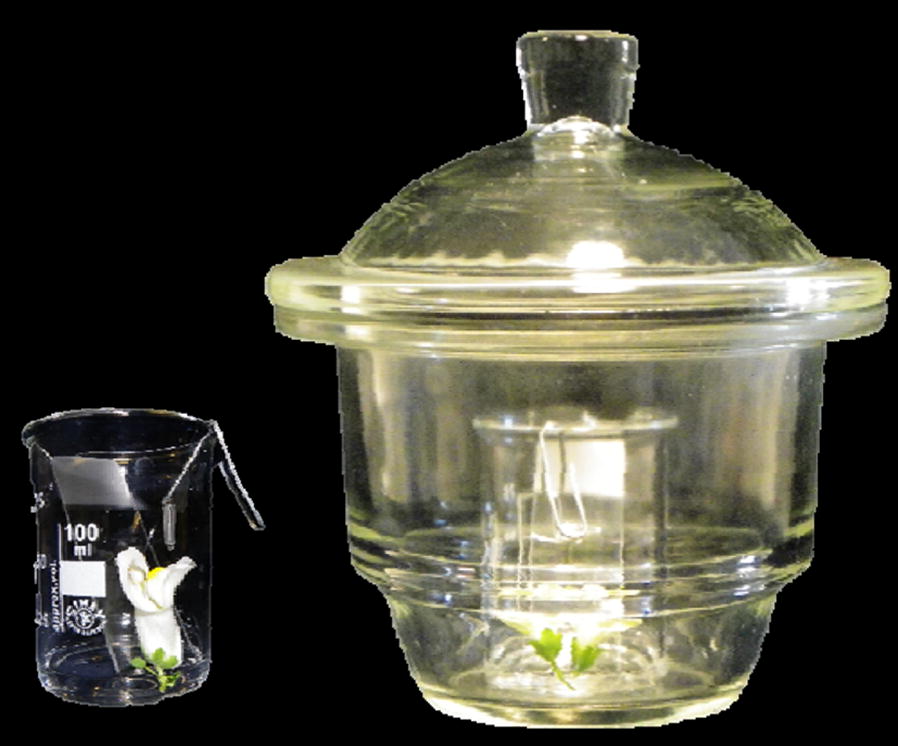
Sampling system for VOCs in HSSE

We used completely developed 3–4 days old flowers of the *Antirrhinum majus* inbred line 165E [8, 24] in order to generate the raw data which were then used to compare semi-quantification methods. Additionally, a flower scent profile was generated for *Petunia x hybrida* line Mitchell. The sampling system consisted in flowers placed inside a beaker with 4 ml of 5% sucrose in distilled water, supported by a glass slide, and a stir bar was attached to the border of the beaker with a stainless-steel paperclip. The beaker was then placed in a 2-l desiccator (Fig. 1).

Flowers for CG-MS analysis were kept under conditions of 12 h light and 12 h dark at 23 °C and 18 °C, respectively, in a growth chamber (Sanyo MRL 350). In case of *A. majus*, stir bars sampled the floral volatiles of 3 flowers in 3 different desiccators during 12 h of light or 12 h of dark periods. In case of *Petunia x hybrida*, stir bars sampled floral volatiles for 4 or 24 h, sampling times applied in circadian rhythm studies [25]. The VOC profile of *A. majus*, is based on compounds which appeared unanimously in the day and night replicas (Table 1). Contaminants were identified and omitted in subsequent analyses.

**Table 1:** Chromatographic parameters for *A. majus* VOCs analysed in column HP5 MSVi

Retention time	Compound	CAS	LRI	LRI bibliography	Reference
2.23	Methyl 2-methylbutanoate	868-57-5	805	774	[26]
5.58	β-myrcene	123-35-3	996	991	[27]
6.62	Ocimene	6874-10-8	1044	1038	[28]
7.19	Acetophenone	98-86-2	1071	1065	[27]
7.77	Methyl benzoate	93-58-3	1099	1091	[27]
7.96	Nonanal	124-19-6	1107	1104	[29]
9.03	Acetophenone, 2’-hydroxy	118-93-4	1167	1160	[26]

We used 10 mm long Twisters™ (Gerstel, Mülheim an der Ruhr, Germany) (stir bars), covered with a 0.5 mm film of polydimethylsiloxane (PDMS). We also tested dual-phase stir bars (ethylene glycol and silicone) (Gerstel, Mülheim an der Ruhr, Germany). Both types of stir bars were conditioned for adsorption according to manufacturer indications.

Compounds adsorbed by the stir bars were analysed by GC–MS in a gas chromatograph HP-6890N coupled to a 5975 mass spectrometer (Agilent Technologies, Palo Alto, USA) combined with a TDU and cooling injector system (CIS4) (Gerstel, Mülheim an der Ruhr, Germany).

Desorption was carried out by heating from an initial temperature of 40° to 250 °C at 100 °C min^−1^ with 5 min hold time on splitless mode. Desorbed compounds were captured in a cool trap at − 100 °C. This process was automated by using a multipurpose sampler MPS2XL (Gerstel, Mülheim an der Ruhr, Germany).

Chromatographic separation was done in a HP5MS-UI column (Agilent Technologies, Palo Alto, USA) with helium as gas carrier in constant pressure mode and split ratio 1:50. Initial temperature was 50 °C, increasing at a ratio of 5 °C min^−1^ until 70 °C held 1 min. In the next step, temperature was increased until 240 °C at 10 °C min^−1^ held for 15 min.

The mass spectrometer operated at 70 eV ionization voltage. Source and quadrupole temperatures were 230 and 150 °C, respectively. Mass range was 30.0 to 450.0 uma at 4 scan/s. MSD transfer line was maintained at 280 °C.

We used ChemStation software (version E.02.02 SP1, Agilent Technologies, Palo Alto, USA) to acquire chromatograms. Compounds were qualitatively identified by comparison with mass spectral database Willey10th-NIST11b (Agilent Technologies, Wilmington, USA), considering match qualities above 90%. We used ocimene, acetophenone, methyl benzoate and methyl cinnamate (Sigma-Aldrich, W353901, 42163, 18344 and 96410, respectively) as standards. Methanol was used as solvent for dilution of standards (Panreac, 361091). Linear retention indexes (LRI) were calculated as a parameter for identifying compounds by comparing with retention times (RT) of C8-C20 alkanes (Sigma Aldrich, 04070), analysed under the same chromatographic conditions (Table 1) [30].

### Semi-quantitative methods of VOCs analysis

We analysed raw data using: (1) external calibrators obtained by adding standards to the sampling system, (2) external calibrators obtained by liquid addition to stir bars, (3) calculation of integrated peak area·gFW^−1^, (4) calculation of normalized peak area·gFW^−1^, (5) semi-quantification based on internal standard, (6) semi-quantification based on external calibrator area using the NearestRT *n*-alkane and (7) percentage calculation.

#### Method 1. Calibration curves obtained by adding standards to the sampling system

We used two different methods to apply standards to the sampling system, method 1A generates calibration curves by standard addition, whereas method 1B generates external calibration curves.

In the first case (method 1A), a mixture of standards (25, 50 and 100 mg/L) including ocimene, acetophenone, methyl benzoate and methyl cinnamate was added directly to the sucrose solution together with four individual *Antirrhinum* flowers of the same plant, distributed in four desiccators. This experiment was duplicated.

In the second case (method 1B), standards with different concentrations of ocimene, acetophenone, methyl benzoate and methyl cinnamate were added directly to the sucrose solution without flower (Table 2). The concentration ranged from 11.25 to 900 mg/L (ocimene) and from 50 to 1000 mg/L (acetophenone, methyl benzoate and methyl cinnamante). A total of 6 standard mixtures with 3 replicas were applied to different sampling systems. Calibration curves were obtained by using Chemstation. We used the total integrated peak area of each compound for further semi-quantification and the calibrator approaches A) and B) as described below.

**Table 2:** External calibration curves carried out in headspace and by liquid addition of standards to stir bars

External calibration curve	Standard	Retention time	Calibration curve	r^2^	Unit
Standards to sampling system (1B)	Ocimene (E)	6.60	6.299·10^8^ ×	0.98	mg
	Ocimene (Z)	6.80	1.196·10^9^ ×	0.98	
	Acetophenone	7.20	5.247·10^8^ ×	0.96	
	Methyl benzoate	7.77	1.345·10^9^ ×	0.96	
	Methyl cinnamate	12.61	2.891·10^9^ ×	0.99	
Standards to stir bars (2)	Ocimene (E)	6.54	3.424·10^6^ × − 9.325·10^4^	0.99	µg
	Ocimene (Z)	6.81	8.318·10^6^ × − 1.397·10^5^	0.99	
	Acetophenone	7.18	1.052·10^7^ × − 2.693·10^5^	0.99	
	Methyl benzoate	7.78	1.181·10^7^ × − 1.009·10^5^	0.99	
	Methyl cinnamate	12.66	1.762·10^7^ × − 5.245·10^5^	1	

#### Method 2. External calibration curves obtained by adding standards to stir bars

The same aforementioned standards were used in order to obtain calibration curves by adding liquid aliquots directly to stir bars. The concentration of ocimene ranged from 25 to 500 mg/L while acetophenone, methyl benzoate and methyl cinnamate ranged from 50 to 500 mg/L. A total of 5 standard mixtures with 3 replicas were applied to different stir bars in an injection volume of 0.5 µl. Calibration curves were obtained by using Chemstation (Table 2).

As in case of method 1B, we used the total integrated peak area of each compound for furthersemi-quantification, and the calibrator approaches (A) and (B) as described below:

(A) NearestRT: semi-quantifying those compounds lacking standards by using as calibration curve the nearest component among ocimene, methyl benzoate and acetophenone. In this case for instance, we have semi-quantified nonanal (RT 7.958 min) with the methyl benzoate (RT 7.773) calibration curve.
(B) Single calibrator: using a single calibration curve (ocimene, methyl benzoate or acetophenone) for quantifying all the compounds on the scent profile of *A. majus*. For instance, using methyl benzoate calibration curve to semi-quantify the emission of each compound of interest: methyl 2-methylbutanoate, β-myrcene, ocimene, acetophenone, methyl benzoate, nonanal and acetophenone 2-hydroxy.

#### Method 3. Calculation of peak area per fresh weight

The relative abundance of compounds was expressed as the total integrated area of each compound divided by the FW of the sample.

#### Method 4. Calculation of normalized peak area per fresh weight

Normalization of peak areas of each compound was done by using 1-phenylethanol (RT 7.096, Sigma-Aldrich, P13800) as an internal standard by adding 10 µL (0.1%) to the sucrose solution during the flower scent analysis. The normalized peak areas of all compounds were calculated by dividing their total integrated peak area by the integrated peak area of the internal standard.

#### Method 5. Semi-quantification based on a single internal standard peak

Semi-quantification was done by extrapolating the area of 10 µL (0.1%) of 1-phenylethanol (added to each sample) to the integrated area of every compound in the profile.

#### Method 6. Semi-quantification based on the NearestRT *n*-alkane

We added 1 µl of *n*-alkane standard solution C8-C20 (Sigma Aldrich, 04070) to a stir bar. The default concentration of each *n*-alkane in the solution was 40 mg/L. Each *n*-alkane was used as an external calibrator of the NearestRT compound of interest (Table 3). Integrated areas of the NearestRT *n*-alkane were used for semi-quantification by extrapolating the area of 40 mg/L of the nearest *n*-alkane to the integrated area of the compound of interest.

**Table 3:** Alkanes used for semi-quantifying compounds of interest

RT compound	Compound	RT alkane	Alkane used for semi-quantification
2.23	Methyl 2-methylbutanoate	2.325	Octane
5.58	β-myrcene	5.820	Decane
6.62	Ocimene	5.820	Decane
7.19	Acetophenone	7.954	Undecane
7.77	Methyl benzoate	7.954	Undecane
7.96	Nonanal	7.954	Undecane
9.03	Acetophenone, 2′-hydroxy	9.797	Dodecane

#### Method 7. Percentage analysis

In order to calculate the percentage of VOCs from raw data profiles, we selected 7 compounds emitted both during day and night by *Antirrhinum* flowers. The sum of emission of these 7 compounds was considered as 100%.

### Statistics

We performed statistical analysis on the raw data for every semi-quantification method described above. Raw data generated during the day were used to analyse proportional variations of each compound within the scent profile. Second, we retrieved the differences in the day vs. night emission of the compounds of interest. Levene’s, one-way ANOVA and Tukey’s tests were performed by using R (Rcmdr package).

## Results

### Comparison of stir bars and sampling time in HSSE

**Fig. 2:**
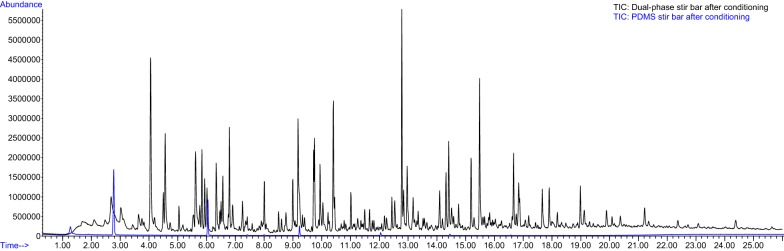
Comparison of commercially available stir bars: blank chromatograms of dual-phase and PDMS stir bars after conditionings according to the indications of manufacturer

We tested two types of stir bars commercially available, the PDMS and dual-phase. Based on the adsorption characteristics, dual-phase should perform better than PDMS adapted to HSSE. However, we obtained a very high background noise by using the dual-phase stir bars (Fig. 2), that may completely cover true signals from biological tissues. Our results indicate that dual-phase stir bars are not suitable to sample complex matrices, such as flowers, due to the high noisy background introduced (Fig. 2). We therefore performed the rest of the measurements and experiments with PDMS bars.

**Fig. 3:**
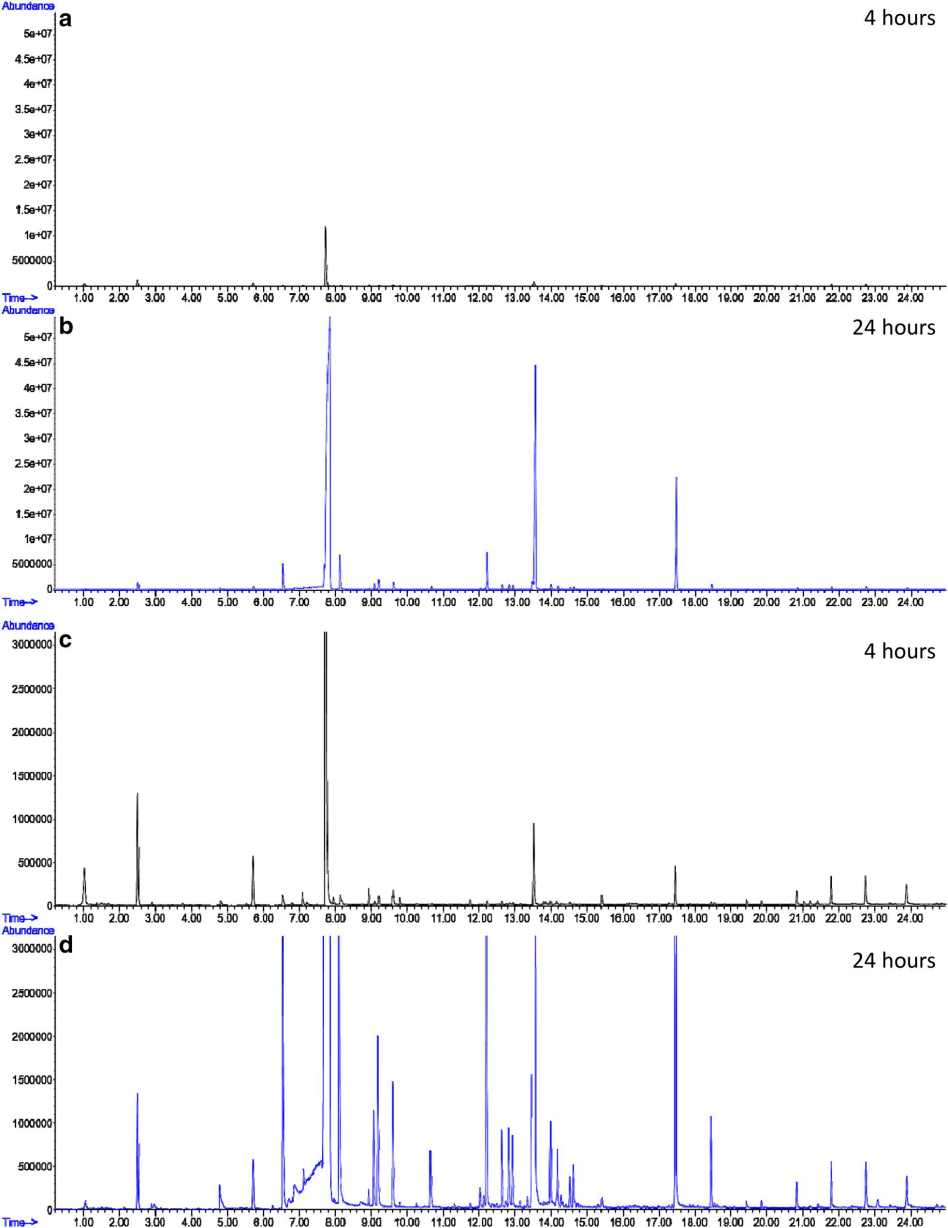
Effect of sampling time on the identification of VOCs. Comparison of floral scent chromatograms of Petunia after 4 h (**a**, **c**) and 24 h (**b**, **d**) of sampling. **c**, **d** are close ups of chromatograms (**a**, **b**). Scales of **a**, **b**, and **c**, **d** are the same for comparison

Most plants emit their floral scent preferentially at certain times of the day. Plants emitting mostly during the day include rose, narcissus or *Antirrhinum*, while plants with preferential night emission include *Petunia* or *Nicotiana* [11, 31, 32]. As sampling frequency plays a role in the detection of circadian rhythms, we tested the effect of sampling time on VOC profiles analysed by HSSE. We sampled a Petunia flower for 4 and 24 h. Figure 3 shows a chromatogram after 4 h of sampling compared to 24 h of sampling under identical conditions. Sampling periods did not seem to affect greatly the acquisition of major compound (Fig. 3a, b). However, the total number of compounds as well as abundances of VOCs were notably affected by sampling time (Fig. 3c, d). Minor VOCs such as benzyl acetate (CAS 140-11-4, RT 9.087, quality 97), benzyl 2-methylbutyrate (CAS 56423-40-6, RT 12.625, quality 97) and (Z)-isoeugenol (CAS 5912-86-7, RT 12.939, quality 98) were detected only in samplings of 24 h. Our results show that sampling time needs to be taken into account when characterising VOC profiles of plant species based on HSSE.

### Comparison of semi-quantification methods

#### Method 1. Calibration curves obtained by adding standards to the sampling system

**Fig. 4:**
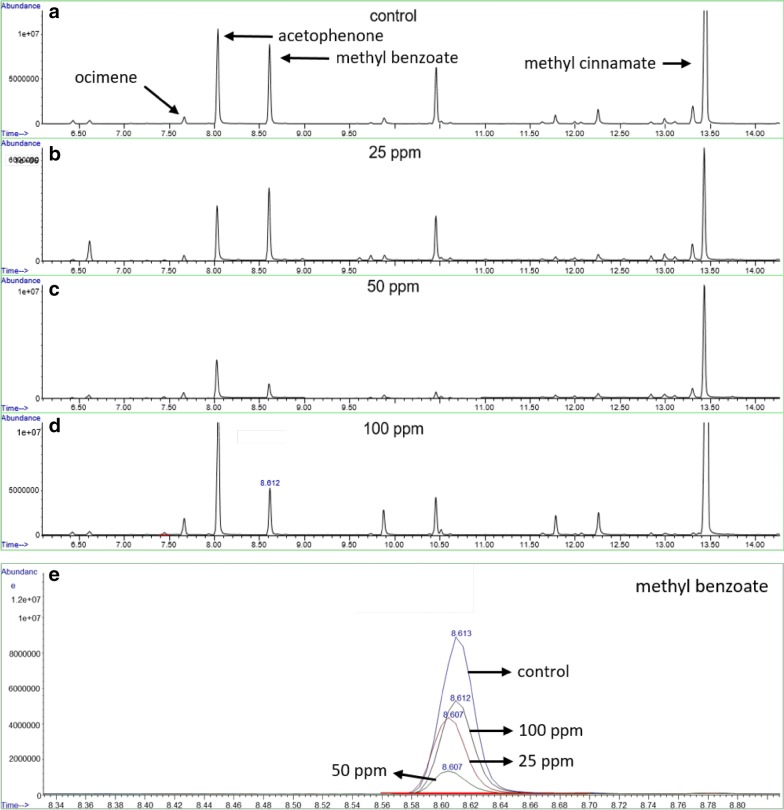
VOC chromatograms of *A. majus* flowers after adding standards to the headspace. **a** Chromatogram of a control flower. **b**–**d** chromatograms after adding 25, 50 and 100 mg/L of the standards. The standards ocimene, acetophenone, methyl benzoate and methyl cinnamate were added directly to the headspace containing flowers from the same plant at the same developmental stages. **e** Overlaid view of the methyl benzoate peaks from chromatograms (**a**–**d**) (RT 8.6)

The feasibility of using calibration curves in the headspace was tested using two approaches. In case of method 1A, we intended to quantify four compounds, ocimene, methyl benzoate, acetophenone and methyl cinnamate by adding mixtures of these standards together with the flower. We used flowers of the same plant for each concentration in order to obtain the corresponding calibration curves and eliminate the matrix effect (Fig. 4a–e). We expected a linear evolution of peak areas for each compound, but instead we observed high variation between flowers, indicating that this calibration procedure is not applicable based on the high natural variability among flowers of one plant. This problematic is illustrated in the chromatogram of a control flower that emits more methyl benzoate than those supplemented with standard solutions (Fig. 4e), indicating that this method does not allow an appropriate quantification.

**Fig. 5:**
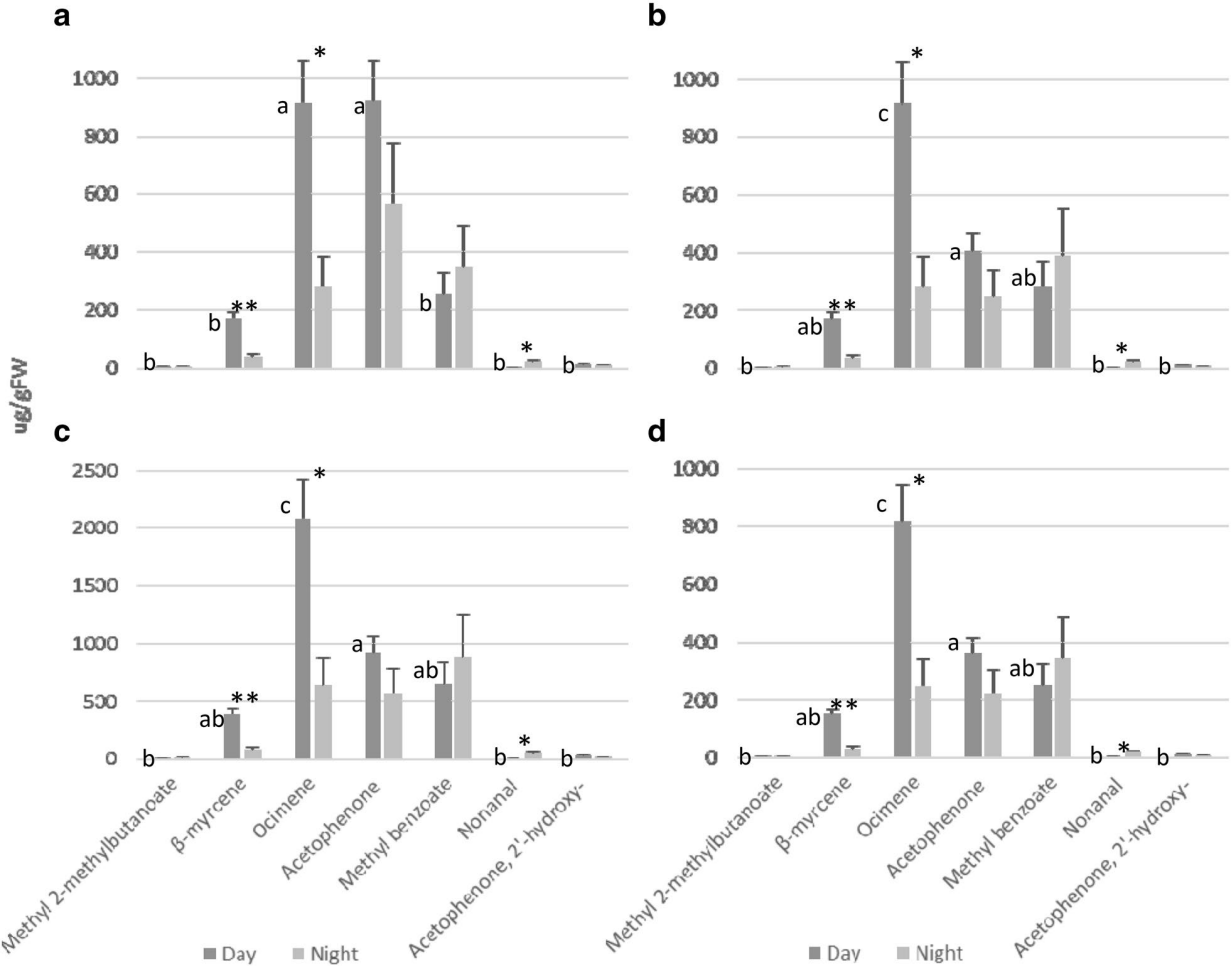
Semi-quantification of VOC compounds emitted by *A. majus flowers* via external calibration curves carried out in the sampling system without flowers. **a** Application of the NearestRT calibration curve. **b** Application of single calibrator ocimene. **c** Application of single calibrator acetophenone. **d** Application of single calibrator methyl benzoate. Semi-quantifications were applied to raw data from day and night. Figures show the mean values in µg gFW^−1^ of three samples for each compound and error bars indicate the standard error. Different letters indicate statistical differences between compounds during the day. Asterisks indicate statistical differences of individual compound between day and night

In case of method 1B, we semi-quantified our raw data of *A. majus* VOCs with external calibration curves carried out by adding standards to the sampling system without flowers (Table 2) using the approaches NearestRT (semi-quantifying those compounds lacking standards by using the calibration curve of the nearest component) and single calibrator. Results show that quantities obtained by the NearestRT procedure or by a single calibrator ocimene or methyl benzoate (Fig. 5a, b, d) peaked at over 1000 µg gFW^−1^. In contrast, in case of acetophenone as single calibrator, quantities were over a 40% higher (Fig. 5c). Concerning differences in the scent profile during the light period, we found two patterns. While single calibrators (Fig. 5b–d) resulted in an identical statistical difference pattern with significant differences among acetophenone and ocimene (Tukey *p* value 0.0017), acetophenone was not different from ocimene (Tukey *p* value 1) in case of the NearestRT calibration (Fig. 5a, Additional file 1: Tables S1, S2 and S3). Differences in VOCs between day and night using different calibrator approaches were identical with ANOVA *p* value of 0.0057, 0.024 and 0.017, respectively, for β-myrcene, ocimene and nonanal (Fig. 5, Additional file 1: Tables S4 and S5).

#### Method 2. External calibration curves carried out by liquid addition to stir bars

**Fig. 6:**
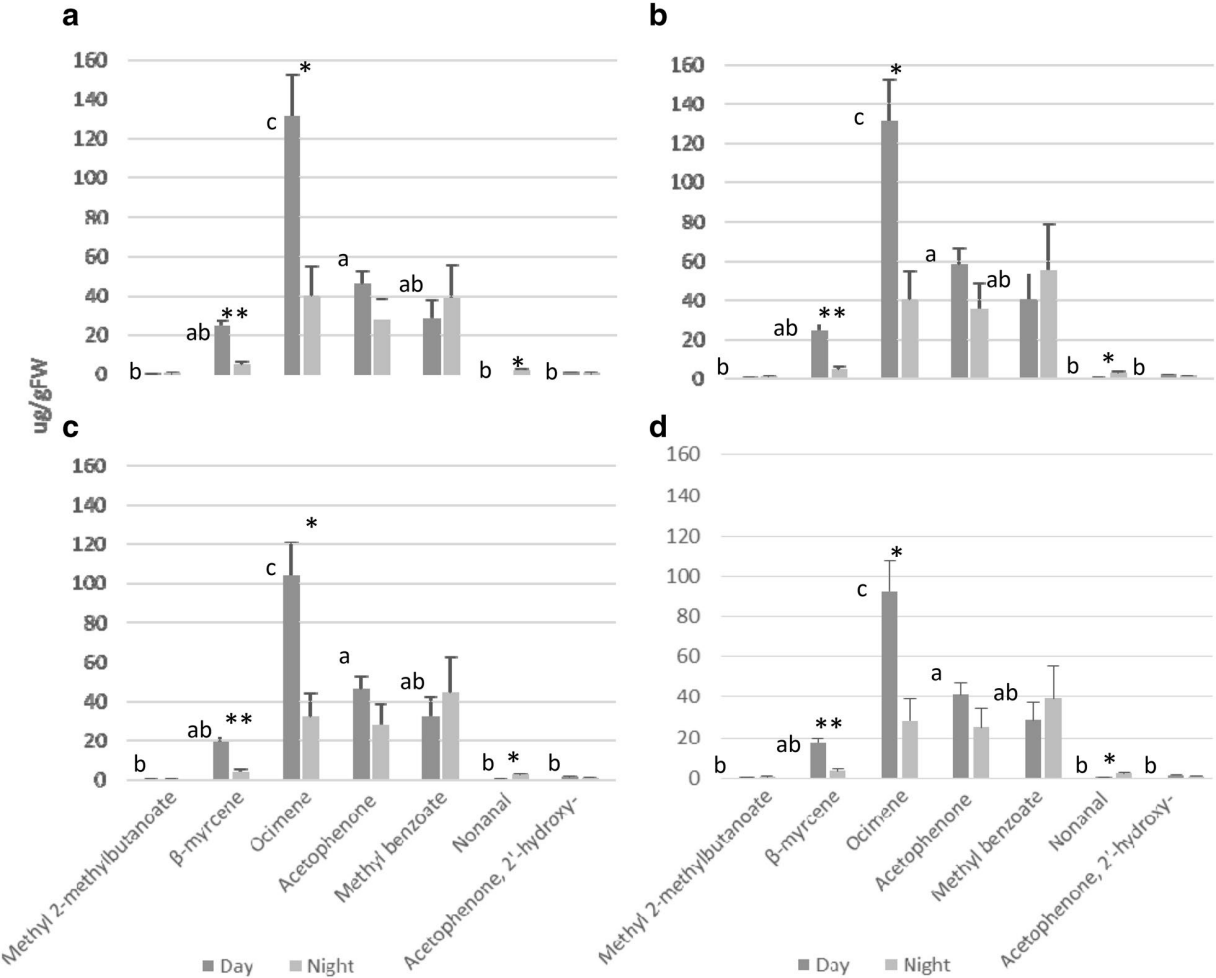
Semi-quantification of VOC compounds emitted by *A. majus flowers* via external calibration curves obtained by adding standards to stir bars. **a** Application of the NearestRT calibration curve. **b** Application of single calibrator ocimene. **c** Application of single calibrator acetophenone. **d** Application of single calibrator methyl benzoate. Semi-quantifications were applied to raw data from day and night. Figures show the mean values in µg gFW^−1^ of three samples for each compound and error bars indicate the standard error. Different letters indicate statistical differences between compounds during the day. Asterisks indicate statistical differences of individual compound between day and night

Following the same key principles applied to the prior way of semi-quantifying (NearestRT and single calibrator), we semi-quantified our raw data of *A. majus* VOCs emitted during the light and dark periods by using external calibration curves obtained by adding standards directly to stir bars (Table 2). We first analysed statistical differences in VOC semi-quantification during a collection period of 12 h (day) (Fig. 6, Additional file 1: Tables S1, S2 and S3). Our results indicate that total quantities of compounds varied between the different approaches. For instance, ocimene emission during the day ranged from 93 to 132 µg gFW^−1^ depending on the calibration system (Fig. 6a, b). On the other hand, the variance and the statistical significances between compounds were maintained (Fig. 5a–d) as between ocimene and acetophenone with *p* value of 0.00016 in case of the NearestRT and 0.0017 in case of the single calibrators (Tukey’s test). When analysing the differences in VOC profiles between day and night, statistical significant patterns were maintained among calibrator approaches with ANOVA *p* value for β-myrcene, ocimene and nonanal (Fig. 6, Additional file 1: Tables S4 and S6) of 0.0057, 0.024 and 0.017, respectively.

Our main conclusion regarding external calibration curves is that using a single calibrator provides identical statistical results irrespective of the compound or external calibrator chosen. In contrast, using the NearestRT, may be subject to changes depending on selected compounds.

#### Method 3. Peak area per gram of FW

**Fig. 7:**
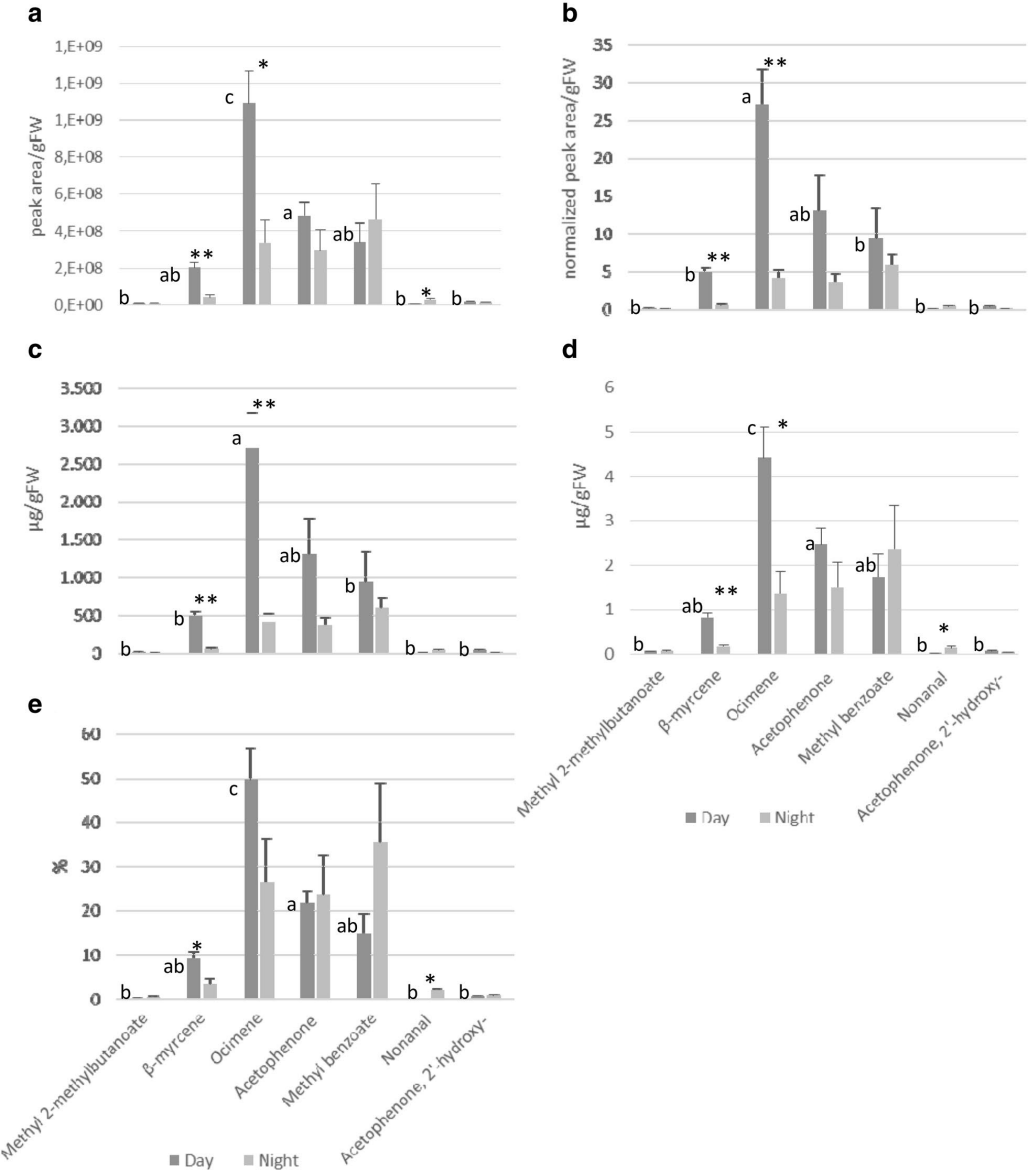
Semi-quantifications of *A. majus* flowers profiles by using different methodologies based on: **a** total integrated peak area **b** normalized area, **c** single internal standard peak (1-phenylethanol), **d** NearestRT *n*-alkane abundance and **e** percentage abundance. Semi-quantifications were applied to raw data from day and night. Figures show the mean values of three samples for each compound and error bars indicate the standard error. Different letters indicate statistical differences between compounds during the day. Asterisks indicate statistical differences of individual compound between day and night

An alternative method of reporting the relative abundance of compounds is expressing the total integrated area of each compound divided by the FW of the sample (Fig. 7a). We evaluated the statistical difference pattern among the relative amounts of compounds of the day-light profile and did not find differences compared to the previously presented methods. For instance, the difference in emission between ocimene and acetophenone was significant with a Tukey’s *p* value of 0.0017, identical to the value obtained by single calibrators (Additional file 1: Tables S1, S2 and S3). Similarly, when analysing the emission of each compound during day and night, statistical significances were identical to those found for the two semi-quantification approaches based on calibration curves. ANOVA *p* value for the differences in emission of β-myrcene, ocimene and nonanal during day and night were: 0.0057, 0.024 and 0.017, respectively (Additional file 1: Tables S4 and S7).

#### Method 4. Normalized peak area per gram of FW

Normalizing gas-chromatographic data is understood as a way of decreasing the experimental error. For this normalization procedure, it is necessary to add a certain quantity of an internal standard not emitted by the sample.

Concerning the scent profiles emitted during the day, statistical results varied slightly compared to the methods applying the total integrated peak areas (see above). According to Tukey’s test, acetophenone and ocimene emission were not statistically different (*P* 0.056) (Fig. 7b, Additional file 1: Tables S1, S2 and S3). Likewise, comparative analysis of the emission between day and night resulted in a different statistical result compared to the previously described semi-quantitative approaches. In this case, the emission of β-myrcene and ocimene were significantly different (*P* 0.0022 and 0.0092), but nonanal was not (*P* 0.072) (Fig. 7b, Additional file 1: Tables S4 and S7).

#### Method 5. Semi-quantification based on internal standard abundance

A quite extended method of semi-quantifying the emission of compounds is to extrapolate the integrated area of the added internal standard to the integrated area of any compound in the chromatogram.

When applying this method, mean quantities of every compound in the scent profile increased considerably compared to the semi-quantitative methods based on calibration curves. Regarding the statistical difference pattern among scent compounds emitted during the day as well as between day and night, results were identical to the normalized peak area method. During the day, a statistical difference between acetophenone and ocimene emission was lacking (Tukey *p* value 0.056), (Fig. 7c, Additional file 1: Tables S1, S2 and S3). Similarly between day and night, the emission of β-myrcene and ocimene (*P* 0.0022 and 0.0092) was statistically significant, but not of nonanal (*P* 0.072) (Fig. 7c, Additional file 1: Tables S4 and S7).

#### Method 6. Semi-quantification based on the nearest *n*-alkane

Following the key principle that similar compounds elute at similar retention times, we used *n*-alkanes to semi-quantify our raw data. The nearest in retention time *n*-alkane (NearestRT alkane) has been used to semi-quantify the compounds of interest. As an example, β-myrcene and ocimene (RT 5.57 and 6.615 min, respectively) have been semi-quantitated by using decane (RT 5.82 min) abundance (Table 3).

The analysis of the scent profile during the day (Fig. 7d, Additional file 1: Tables S1, S2 and S3) indicates that quantities of compounds varied largely when compared to the rest of approaches studied. Regarding the statistical difference pattern among compounds, these differences were similar to those obtained in semi-quantifications based on the methods using total peak areas (Fig. 7d), with a statistical difference between acetophenone and ocimene of *p* value 0.025 (Tukey). Similarly, differences between day and night emission of β-myrcene, ocimene and nonanal were statistically significant (ANOVA *p* value 0.0058, 0.024 and 0.018, respectively) as already observed for the methods based on total peak areas (Fig. 7d, Additional file 1: Tables S4 and S7).

#### Method 7. Percentage analysis

A convenient way to show the relative abundance of VOCs within a scent profile is to express the data in percentages. We found that the statistical significance pattern among the compounds emitted during the day was similar to the NearestRT *n*-alkane method as well as those based on total peak area. We found a statistical difference between acetophenone and ocimene of Tukey´s *p* value 0.00062 (Fig. 7e, Additional file 1: Tables S1, S2 and S3). Concerning statistical differences between day and night, β-myrcene and nonanal showed significant difference whereas ocimene did not. ANOVA *p* value for β-myrcene, ocimene and nonanal were: 0.035, 0.132 and 0.0043, respectively (Fig. 7e, Additional file 1: Tables S4 and S7).

## Discussion

Most studies on VOCs emission concentrate on one to several compounds emitted by a set of samples [33–36], whereas fewer studies focus on entire scent profiles [8, 37–39]. The reason lies in the complexity of this type of analyses. Firstly, it requires the selection of an appropriate chromatographic method which allows to detect all emitted VOCs [18, 40]. Secondly, many variables influence the quantity of VOCs released from the samples, such as light, temperature [37], physiological status of tissues [34] and even air pollution [41], leading to a high variability in the collected data [16]. Many investigators therefore concentrate on a few major compounds, which are constitutively emitted by their research objects, rather than dealing with minor compounds that may or may not be found due to reasons such as natural variability, VOC contamination or VOC emission in a circadian fashion [42]. On the other hand, studying the entire scent profile may give insight in very complex phenomena such as interactions between plants and pollinators [40, 43, 44], volatile perception related to disease detection [7, 45–47], or pheromone signalling [48, 49]. Aromas are phenotypic traits that identify species as a result of evolutionary selection. Establishing which compounds and in which proportion contribute to scent profiles, is a determinant issue for characterizing species as well as for evaluating their effects over different taxa [5, 8, 40, 50].

### Sampling time and stir bars for HSSE

The use of stir bars in HSSE analysis requires an atmosphere in equilibrium, sealed and isolated. PDMS coated stir bars preferably adsorb non-polar compounds. VOCs with different volatility will therefore be adsorbed differentially according to their chemical features [22, 51]. As shown in this work, the number of compounds identified is directly related to the time samples are exposed to the stir bars. This has important implications as VOCs emissions are under circadian regulation in many plants including *Antirrhinum* and *Petunia* [11, 32]. As sampling time plays a key role in the detection of rhythms, and sampling time of 4 h is the minimum required [25], our results indicate that increasing the sampling density will necessarily result in fewer minor VOCs identified.

Additionally, stir bars in headspace show a very high relative standard deviation [52]. Variability using stir bars results from two factors: changes in temperature and the matrix effect. The matrix effect is caused by the equilibrium conditions between matrix/headspace and the headspace/PDMS of the stir bar [52, 53]. In our study, temperature was under strict control and its effects on variability can be excluded. However, the matrix effect is difficult to control when complex matrices are used, such as plant tissues or organs.

### Normalized area vs total peak area

Semi-quantification of compounds using normalized peak area is based on the usage of a specific amount of an internal standard. However, the standard may have chemical characteristics different to the compounds emitted by the sample and therefore may be adsorbed differently by the stir bar. This may cause miscalculations of quantities of VOCs emitted by samples. The use of dual-phase stir bars may improve detection of volatiles, because they more effectively recover polar analytes [54]. However, some drawbacks have been reported [44] and the degree of noise background introduced by these stir bars impedes the identification of minor VOCs.

### Comparison of NearestRT approaches

Based on the key principle that chemically similar compounds elute at similar retention times, we used calibrators of compounds commonly found in the scent profile of *A. majus* as well as *n*-alkanes, which elute at similar retention times as the sample´s compounds, for semi-quantification. Both VOC quantities and proportions varied among the different NearestRT approaches, independent of whether calibrators were added to the headspace or as liquid to stir bars. To our knowledge, this is the first time that *n*-alkanes have been reported for semi-quantifying VOCs as external calibrators. While the NearestRT approach has been applied in combination with internal standards [15, 21], its usage with external calibrators is not documented. We show that the NearestRT approaches presented here are equally valuable for scent profiling.

### Advantages and disadvantages of the studied methodologies

We compared the effect of semi-quantification methods within scent profiles (day time samples) and between scent profiles (day and night time samples). The volatile proportions obtained by different semi-quantification methods, both on intra-sample and inter-sample data, even so not being identical clearly showed a similar trend. These small proportional discrepancies caused, in some cases, statistical variations. Nevertheless we consider that these small variations do not compromise any of the methods studied.

As shown in this work, several valid approaches exist for analysing GC–MS data using HSSE and TICs in terms of semi-quantification. All these procedures provide general, yet accurate, information about profile features. Nevertheless, each of the methodologies analysed here beholds specific advantages and disadvantages related to accuracy, experimental variability, acceptance and retrieved quantities (Table 4).

**Table 4:** Overview of advantages and disadvantages of the semi-quantifying approaches

Advantages	Disadvantages
Method 1B. (*) External calibration curves obtained by adding standards to the sampling system	
High accuracy due to identical sampling conditions between external calibrators and samples	Calibration curves are valid only for the specific sampling conditions (i.e. time or headspace volume)
Method 2. (*) External calibration curves obtained by adding standards to stir bars	
Calibration curves are valid independent of sampling conditions	Lower accuracy due to different sampling conditions between external calibrators and samples
*NearestRT	
High accuracy due to the usage of chemically similar compounds for semi-quantification	Several calibrators along the chromatogram need to be used
*Single calibrator	
Statistical significance of the data is consistent, indicating that any calibrator is valid	A certain level of inaccuracy may result from a lack of chemical similarity between calibrator and sample VOCs
Method 3. Peak area/g fresh weight	
It indicates the relative abundance among VOCs	There is no magnitude
Method 4. Normalized peak area/g fresh weight	
Generally accepted as a precise mean to analyze relative abundance among VOCs	Bias due to differential stir bar adsorption between the internal standard and certain kinds of VOCs There is no magnitude
Method 5. Single internal standard peak	
Generally accepted as a precise mean for semi-quantification	Bias due to differential stir bar adsorption between the internal standard and certain kinds of VOCs
Method 6. NearestRT n-alkane	
High accuracy due to the usage of chemically similar compounds for semi-quantification	A certain level of inaccuracy may result from a lack of chemical similarity between calibrator and sample VOCs
Method 7. Percentage analysis	
Generally accepted as a precise mean to analyze relative abundance among VOCs	There is no magnitude

The asterisks indicate that NearestRT and single calibrator were used in method 1B and method 2

Regarding accuracy, the use of the NearestRT approach (methods 1B, 2 and 6) could be considered more appropriate than the single calibrator approach (methods 1B and 2) and internal standard abundance (method 5), because chemically more similar compounds are used.

Concerning experimental variability, the outcome of external calibration curves in headspace is affected by the experimental conditions, like sampling time or volume in the headspace container, and need to be adapted to those used for VOC sampling. In contrast, this problematic does not occur when applying methods based on liquid addition directly to stir bars (method 2 and 6).

Quite popular methodologies are those based on internal standard abundance [10, 19, 20, 40] and percentages [14, 31, 55, 56], because they accurately reflect relative abundances. We show here that less common methods like total peak areas or normalized areas of compounds [8, 14] are valid alternatives for this purpose. However, none of these two approaches have a magnitude and understanding abundances is not straightforward. The single calibrator approach has been reported, but with some methodological differences [57] and no publications are available expressing results as total peak areas·gFW^−1^.

Regarding VOC quantities, the only informative methods are: external calibration curves from headspace (method 1B), external calibration curves from liquid addition to stir bars (method 2), semi-quantification based on a single internal standard peak (method 5) and semi-quantification based on the NearestRT *n*-alkane. However, quantities may vary depending on the methods and this difference should be taken into account especially when comparing results from different publications.

Despite all the divergences, all methods can be considered as reliable means of analysing scent profiles.

## Conclusions

Two main conclusions can be drawn from this study. First, semi-quantification by standard addition is not a feasible method in sets of samples with a high biological variability, as in case of flowers. Secondly, any of the methodologies studied adequately reflects the relative proportion of VOCs when screening volatile metabolomes.

From our point of view and concerning the plant scent community, a general methodological consensus would be desirable in order to ease the comparison of data.

## Additional file

**Additional file 1.** Statistical analysis of different semiquantitative methods.
