# Supplementary material for: A comparison of semi-quantitative methods suitable for establishing volatile profiles
Source: Plant Methods. 2018 Aug 9;14:67. doi: 10.1186/s13007-018-0335-2 (PMC6083509; doi:10.1186/s13007-018-0335-2)
Supplement: Supplementary file 1 — Additional file 1. Statistical analysis of different semiquantitative methods. [file 13007_2018_335_MOESM1_ESM.docx]

# Additional file

Table S1. Levene´s test for homoscedasticity of all *A. majus* flowers VOCs emitted during the day, applying the different semi-quantification methods.

| Method (nr.) | F value | P-value |
| --- | --- | --- |
| Single calibrator: standards to sampling system (1B) and to stir bars (2); total peak area·g FW (3) | 1.017 | 0.4534 |
| NearestRT – standards to sampling system (1B) | 1.0642 | 0.428 |
| NearestRT – standards to stir bars (2) | 1.1201 | 0.3996 |
| Normalized peak area·g FW (4); single internal standard peak (5) | 1.6699 | 0.2009 |
| NearestRT *n-*alkane (6) | 0.9522 | 0.4903 |
| Percentage (7) | 1.572 | 0.2271 |

P values ≤ 0,05 are considered significant. Degrees of freedom: 6.

Table S2. Statistic significance among VOCs for the entire day-time scent profile of *A. majus* flowers according to ANOVA.

| Method (nr) | Calibration curve | Sum Sq | Mean Sq | F-value | P-value | Signif. |
| --- | --- | --- | --- | --- | --- | --- |
| Standards to sampling system (1B) | NearestRT | 3105655 | 517609 | 25.57 | 0.000000884 | *** |
|  | Ocimene | 1947909 | 324651 | 23.15 | 0.00000165 | *** |
|  | Acetophenone | 10120660 | 1686777 | 23.15 | 0.00000165 | *** |
|  | Methyl benzoate | 1540233 | 256705 | 23.15 | 0.00000165 | *** |
| Standards to stir bars (2) | NearestRT | 39212 | 6535 | 26.8 | 0.000000658 | *** |
|  | Ocimene | 40271 | 6712 | 23.15 | 0.00000165 | *** |
|  | Acetophenone | 25177 | 4196 | 23.15 | 0.00000165 | *** |
|  | Methyl benzoate | 19977 | 3330 | 23.15 | 0.00000165 | *** |
| Total peak area·g FW (3) |  | 2,786E+21 | 4,644E+20 | 23.15 | 0.00000165 | *** |
| Normalized peak area·g FW (4) |  | 1749.9 | 291.65 | 11.17 | 0.000119 | *** |
| Single internal standard peak (5) |  | 17498702 | 2916450 | 11.17 | 0.000119 | *** |
| Nearest *n-*alkane (6) |  | 5.50 | 8.031 | 20.43 | 0.00000354 | *** |
| Percentage (7) |  | 5707 | 951.1 | 28.25 | 0.000000472 | *** |

*,**, and *** indicate statistical significances for p values ≤ 0.05, ≤ 0.01 and 0.001, respectively and 6 degrees of freedom.

Table S3. Statistical significance among specific VOCs of the day-time scent profile of *A. majus* flowers according to TukeyHSD test. Statistical significance p-value < 0.05. Bold numbers indicate statistical significances.

| Compounds | Single calibrators: samp. syst. (1B) and stir bars (2); total peak area·g FW (3) | Headspace NearestRT  (1B) | Liquid addition NearestRT  (2) | Normalized peak area·g FW (4); single internal standard peak (5) | NearestRT *n-*alkane (6) | Percent (7) |
| --- | --- | --- | --- | --- | --- | --- |
| Acetophenone, 2-hydroxy--Acetophenone | **1,62E-02** | **2,91E-05** | **4,34E-02** | 9,54E-02 | **7,93E-03** | **5,17E-03** |
| Methyl 2-methylbutanoate-Acetophenone | **1,40E-02** | **2,69E-05** | **4,02E-02** | 8,63E-02 | **6,76E-03** | **5,06E-03** |
| Methyl benzoate-Acetophenone | 8,63E-01 | **7,60E-04** | 8,16E-01 | 9,69E-01 | 7,88E-01 | 7,74E-01 |
| Nonanal-Acetophenone | **1,34E-02** | **2,61E-05** | **3,77E-02** | 8,37E-02 | **6,46E-03** | **4,34E-03** |
| Ocimene-isomers-Acetophenone | **1,72E-03** | 1,00E+00 | **1,56E-04** | 5,61E-02 | **6,16E-04** | **2,45E-02** |
| ß-myrcene-Acetophenone | 2,62E-01 | **2,37E-04** | 6,38E-01 | 4,80E-01 | 1,90E-01 | 7,29E-02 |
| Methyl 2-methylbutanoate-Acetophenone, 2-hydroxy | 1,00E+00 | 1,00E+00 | 1,00E+00 | 1,00E+00 | 1,00E+00 | 1,00E+00 |
| Methyl benzoate-Acetophenone, 2-hydroxy- | 1,47E-01 | 4,22E-01 | 3,82E-01 | 3,74E-01 | 9,96E-02 | 6,95E-02 |
| Nonanal-Acetophenone, 2-hydroxy- | 1,00E+00 | 1,00E+00 | 1,00E+00 | 1,00E+00 | 1,00E+00 | 1,00E+00 |
| Ocimene-isomers-Acetophenone, 2-hydroxy- | **3,70E-06** | **3,12E-05** | **1,20E-06** | **2,62E-04** | **1,10E-06** | **1,15E-05** |
| ß-myrcene-Acetophenone, 2-hydroxy- | 6,71E-01 | 8,11E-01 | 5,60E-01 | 9,21E-01 | 5,65E-01 | 7,59E-01 |
| Methyl benzoate-Methyl 2-methylbutanoate | 1,29E-01 | 3,94E-01 | 3,62E-01 | 3,46E-01 | 8,57E-02 | 6,82E-02 |
| Nonanal-Methyl 2-methylbutanoate | 1,00E+00 | 1,00E+00 | 1,00E+00 | 1,00E+00 | 1,00E+00 | 1,00E+00 |
| Ocimene-isomers-Methyl 2-methylbutanoate | **3,30E-06** | **2,88E-05** | **1,20E-06** | **2,39E-04** | **1,00E-06** | **1,13E-05** |
| ß-myrcene-Methyl 2-methylbutanoate | 6,25E-01 | 7,85E-01 | 5,36E-01 | 9,02E-01 | 5,15E-01 | 7,53E-01 |
| Nonanal-Methyl benzoate | 1,24E-01 | 3,83E-01 | 3,44E-01 | 3,38E-01 | 8,20E-02 | 5,86E-02 |
| Ocimene-isomers-Methyl benzoate | **2,09E-04** | **8,23E-04** | **2,03E-05** | **1,13E-02** | **6,40E-05** | **1,83E-03** |
| ß-myrcene-Methyl benzoate | 8,96E-01 | 9,90E-01 | 1,00E+00 | 9,28E-01 | 8,78E-01 | 5,91E-01 |
| Ocimene-isomers-Nonanal | **3,20E-06** | **2,79E-05** | **1,10E-06** | **2,32E-04** | **9,00E-07** | **1,00E-05** |
| ß-myrcene-Nonanal | 6,12E-01 | 7,73E-01 | 5,15E-01 | 8,95E-01 | 5,00E-01 | 7,06E-01 |
| ß-myrcene-Ocimene-isomers | **3,47E-05** | **2,56E-04** | **1,28E-05** | **1,66E-03** | **1,10E-05** | **9,85E-05** |

Statistical significance p-value < 0.05.

Table S4. Levene´s test for homoscedasticity of all *A. majus* flower VOCs emitted during day and night, applying the different semi-quantification methods.

| Method (nr) | Compound | F-value | P-value |
| --- | --- | --- | --- |
| External calibration curves obtained by adding standards to sampling system (1B) and to stir bars (2); total peak area·g FW (3) | Methyl 2-methylbutanoate | 1.2527 | 0.3257 |
|  | β-myrcene | 0.6882 | 0.4534 |
|  | Ocimene-isomers | 0.0681 | 0.8071 |
|  | Acetophenone | 0.1294 | 0.7373 |
|  | Methyl benzoate | 0.3905 | 0.5659 |
|  | Nonanal | 3.2407 | 0.1462 |
|  | Acetophenone 2-hydroxy | 0.2615 | 0.636 |
| Normalized peak area·g FW (4); single internal standard peak (5) | Methyl 2-methylbutanoate | 0.0089 | 0.9293 |
|  | β-myrcene | 1.7235 | 0.2595 |
|  | Ocimene-isomers | 1.2463 | 0.3268 |
|  | Acetophenone | 1.6384 | 0.2697 |
|  | Methyl benzoate | 1.4091 | 0.3009 |
|  | Nonanal | 1.7789 | 0.2532 |
|  | Acetophenone 2-hydroxy | 1.2246 | 0.3305 |
| NearestRT *n-*alkane (6) | Methyl 2-methylbutanoate | 1.2527 | 0.3257 |
|  | β-myrcene | 0.6466 | 0.4664 |
|  | Ocimene-isomers | 0.0612 | 0.8168 |
|  | Acetophenone | 0.1377 | 0.7294 |
|  | Methyl benzoate | 0.4265 | 0.5493 |
|  | Nonanal | 3.4289 | 0.1377 |
|  | Acetophenone 2-hydroxy | 0.2429 | 0.6479 |
| Percentage (7) | Methyl 2-methylbutanoate | 1,7377 | 0.2579 |
|  | β-myrcene | 0.011 | 0.9214 |
|  | Ocimene-isomers | 0.0294 | 0.8723 |
|  | Acetophenone | 0.6028 | 0.4809 |
|  | Methyl benzoate | 1.381 | 0.3051 |
|  | Nonanal | 3.6833 | 0.1274 |
|  | Acetophenone 2-hydroxy | 0.3369 | 0.5927 |

P values ≤ 0,05 are considered significant. Degrees of freedom: 1

Table S5. Statistic significance between VOCs emitted by *A. majus* flowers during the day and night according to ANOVA using the external calibration curve generated by adding standards to the headspace (Method 1B).

| Calibration curve | Compound | Sum/Mean Sq | F-value | P-value | Signif. |
| --- | --- | --- | --- | --- | --- |
| NearestRT: Ocimene | Methyl 2-methylbutanoate | 0.145 | 0.039 | 0.854 |  |
| NearestRT: Ocimene | β-myrcene | 27614 | 29.13 | 0.0057 | ** |
| NearestRT: Ocimene | Ocimene-isomers | 605483 | 12.57 | 0.0239 | * |
| NearestRT: Acetophenone | Acetophenone | 190105 | 1.974 | 0.233 |  |
| NearestRT: Methyl benzoate | Methyl benzoate | 12952 | 0.321 | 0.601 |  |
| NearestRT: Methyl benzoate | Nonanal | 438.2 | 15.76 | 0.0165 | * |
| NearestRT: Methyl benzoate | Acetophenone 2-hydroxy | 20.031 | 6.392 | 0.0648 |  |
| Single calibrator:  Ocimene | Methyl 2-methylbutanoate | 0.145 | 0.039 | 0.854 |  |
|  | β-myrcene | 27614 | 29.13 | 0.0057 | ** |
|  | Ocimene-isomers | 605483 | 12.57 | 0.0239 | * |
|  | Acetophenone | 36589 | 1.974 | 0.233 |  |
|  | Methyl benzoate | 16380 | 0.321 | 0.601 |  |
|  | Nonanal | 554.2 | 15.76 | 0.0165 | * |
|  | Acetophenone 2-hydroxy | 25.332 | 6.392 | 0.0648 |  |
| Single calibrator:  Acetophenone | Methyl 2-methylbutanoate | 0.75 | 0.039 | 0.854 |  |
|  | β-myrcene | 143472 | 29.13 | 0.0057 | ** |
|  | Ocimene-isomers | 3145882 | 12.57 | 0.0239 | * |
|  | Acetophenone | 190105 | 1.974 | 0.233 |  |
|  | Methyl benzoate | 85106 | 0.321 | 0.601 |  |
|  | Nonanal | 2879 | 15.76 | 0.0165 | * |
|  | Acetophenone 2-hydroxy | 131.62 | 6.392 | 0.0648 |  |
| Single calibrator:  Methyl benzoate | Methyl 2-methylbutanoate | 0.1148 | 0.039 | 0.854 |  |
|  | β-myrcene | 21835 | 29.13 | 0.0057 | ** |
|  | Ocimene-isomers | 478762 | 12.57 | 0.0239 | * |
|  | Acetophenone | 28931 | 1.974 | 0.233 |  |
|  | Methyl benzoate | 12952 | 0.321 | 0.601 |  |
|  | Nonanal | 438.2 | 15.76 | 0.0165 | * |
|  | Acetophenone 2-hydroxy | 20.031 | 6.392 | 0.0648 |  |

*,**, and *** indicate statistical significances for p values ≤ 0.05, ≤ 0.01 and 0.001, respectively and 1 degree of freedom.

Table S6. Statistic significance between VOCs emitted by *A. majus* flowers during the day and night according to ANOVA using the external calibration curve generated by adding standards to stir bars (Method 2).

| Calibration curve | Compound | Sum/Mean Sq | F value | P-value | Signif. |
| --- | --- | --- | --- | --- | --- |
| NearestRT: Ocimene | Methyl 2-methylbutanoate | 0.003 | 0.039 | 0.854 |  |
| NearestRT: Ocimene | β-myrcene | 570.9 | 29.13 | 0.0057 | ** |
| NearestRT: Ocimene | Ocimene-isomers | 12518 | 12.57 | 0.0239 | * |
| NearestRT: Acetophenone | Acetophenone | 472.9 | 1.974 | 0.233 |  |
| NearestRT: Methyl benzoate | Methyl benzoate | 168 | 0.321 | 0.601 |  |
| NearestRT: Methyl benzoate | Nonanal | 5.684 | 15.76 | 0.0165 | * |
| NearestRT: Methyl benzoate | Acetophenone 2-hydroxy | 0.25980 | 6.392 | 0.0648 |  |
| Single calibrator:  Ocimene | Methyl 2-methylbutanoate | 0.003 | 0.039 | 0.854 |  |
|  | β-myrcene | 570.9 | 29.13 | 0.0057 | ** |
|  | Ocimene-isomers | 12518 | 12.57 | 0.0239 | * |
|  | Acetophenone | 756.4 | 1.974 | 0.233 |  |
|  | Methyl benzoate | 338.6 | 0.321 | 0.601 |  |
|  | Nonanal | 11.458 | 15.76 | 0.0165 | * |
|  | Acetophenone 2-hydroxy | 0.5237 | 6.392 | 0.0648 |  |
| Single calibrator:  Acetophenone | Methyl 2-methylbutanoate | 0.00188 | 0.039 | 0.854 |  |
|  | β-myrcene | 356.9 | 29.13 | 0.0057 | ** |
|  | Ocimene-isomers | 7826 | 12.57 | 0.0239 | * |
|  | Acetophenone | 472.9 | 1.974 | 0.233 |  |
|  | Methyl benzoate | 211.7 | 0.321 | 0.601 |  |
|  | Nonanal | 7.163 | 15.76 | 0.0165 | * |
|  | Acetophenone 2-hydroxy | 0.3274 | 6.392 | 0.0648 |  |
| Single calibrator:  Methyl benzoate | Methyl 2-methylbutanoate | 0.00149 | 0.039 | 0.854 |  |
|  | β-myrcene | 283.20 | 29.13 | 0.0057 | ** |
|  | Ocimene-isomers | 6210 | 12.57 | 0.0239 | * |
|  | Acetophenone | 375.2 | 1.974 | 0.233 |  |
|  | Methyl benzoate | 168 | 0.321 | 0.601 |  |
|  | Nonanal | 5.684 | 15.76 | 0.0165 | * |
|  | Acetophenone 2-hydroxy | 0.25980 | 6.392 | 0.0648 |  |

*,**, and *** indicate statistical significances for p values ≤ 0.05, ≤ 0.01 and 0.001, respectively and 1 degree of freedom.

Table S7. Statistic significance between VOCs emitted by *A. majus* flowers during the day and night according to ANOVA using specified methods.

| Method (nr.) | Compound | Sum/Mean Sq | F value | P-value | Signif. |
| --- | --- | --- | --- | --- | --- |
| Total peak area·gFW (3) | Methyl 2-methylbutanoate | 2,0762E+11 | 0.039 | 0.854 |  |
|  | β-myrcene | 3,95E+19 | 29.13 | 0.0057 | ** |
|  | Ocimene-isomers | 8,66E+20 | 12.57 | 0.0239 | * |
|  | Acetophenone | 5,23E+19 | 1.974 | 0.233 |  |
|  | Methyl benzoate | 2,34E+19 | 0.321 | 0.601 |  |
|  | Nonanal | 7,93E+17 | 15.76 | 0.0165 | * |
|  | Acetophenone 2-hydroxy | 3,6236E+13 | 6.392 | 0.0648 |  |
| Normalized peak area·gFW (4) | Methyl 2-methylbutanoate | 0.00563 | 0.565 | 0.494 |  |
|  | β-myrcene | 29.032 | 49.29 | 0.00217 | ** |
|  | Ocimene-isomers | 786.9 | 22.38 | 0.0091 | ** |
|  | Acetophenone | 132.4 | 3.859 | 0.121 |  |
|  | Methyl benzoate | 18.36 | 0.664 | 0.461 |  |
|  | Nonanal | 0.1373 | 5.937 | 0.0715 |  |
|  | Acetophenone 2-hydroxy | 0.09929 | 3.405 | 0.139 |  |
| Single internal standard peak (5) | Methyl 2-methylbutanoate | 56.3 | 0.565 | 0.494 |  |
|  | β-myrcene | 290318 | 49.29 | 0.00217 | ** |
|  | Ocimene-isomers | 7868758 | 22.38 | 0.0091 | ** |
|  | Acetophenone | 1324543 | 3.859 | 0.121 |  |
|  | Methyl benzoate | 183556 | 0.664 | 0.461 |  |
|  | Nonanal | 1373 | 5.937 | 0.0715 |  |
|  | Acetophenone 2-hydroxy | 992.9 | 3.405 | 0.139 |  |
| Nearest *n-*alkane (6) | Methyl 2-methylbutanoate | 1.92307 | 0.039 | 0.854 |  |
|  | β-myrcene | 64523.1079 | 28.99 | 0.00575 | ** |
|  | Ocimene-isomers | 1414008.04 | 12.5 | 0.0241 | * |
|  | Acetophenone | 138300.863 | 1.968 | 0.233 |  |
|  | Methyl benzoate | 56520.1196 | 0.292 | 0.618 |  |
|  | Nonanal | 1943.9696 | 15 | 0.018 | * |
|  | Acetophenone 2-hydroxy | 77.37176 | 6.916 | 0.0582 |  |
| Percentage (7) | Methyl 2-methylbutanoate | 0.08455 | 4.168 | 0.111 |  |
|  | β-myrcene | 51.73 | 9.825 | 0.035 | * |
|  | Ocimene-isomers | 798.5 | 3.555 | 0.132 |  |
|  | Acetophenone | 5.4 | 0.041 | 0.849 |  |
|  | Methyl benzoate | 629.8 | 2.14 | 0.217 |  |
|  | Nonanal | 5.291 | 33.98 | 0.00431 | ** |
|  | Acetophenone 2-hydroxy | 0.028531 | 5.29 | 0.0829 |  |

*,**, and *** indicate statistical significances for p values ≤ 0.05, ≤ 0.01 and 0.001, respectively and 1 degree of freedom.
